# Freshwater salinization and the evolved tolerance of amphibians

**DOI:** 10.1002/ece3.11069

**Published:** 2024-03-12

**Authors:** Rick Relyea, Brian Mattes, Candace Schermerhorn, Isaac Shepard

**Affiliations:** ^1^ Department of Biological Sciences Rensselaer Polytechnic Institute Troy New York USA

**Keywords:** amphibian, evolution, microevolution, sensitivity, toxicology

## Abstract

The increasing salinization of freshwaters is a growing environmental issue as a result of mining, agriculture, climate change, and the application of de‐icing salts in regions that experience ice and snow. Due to narrow osmotic limits, many freshwater species are particularly susceptible to salinization, but it is possible that repeated exposures over time could favor the evolution of increased salt tolerance. Using collected nine populations of larval wood frogs (*Rana sylvatica*) as eggs from ponds and wetlands with close proximity to roads and spanning a wide gradient of salt concentrations. In the first experiment, we used a time‐to‐death experiment to examine the salt tolerance. In a second experiment, we examined whether population differences in salt tolerance were associated with trade‐offs in growth, development, or behavior in the presence of control water or a sublethal salt concentration. We found that populations collected from ponds with low and intermediate salt concentrations exhibited similar tolerance curves over a 96‐h exposure. However, the population from a pond with the highest salt concentration exhibited a much higher tolerance. We also found population differences in growth, development, and activity level among the populations, but these were not associated with population differences in tolerance. In addition, the sublethal concentration of salt had no impact on growth and development, but it did cause a reduction in tadpole activity across the populations. Collectively, these results provide further evidence that some species of freshwater organisms can evolve tolerance to increasing salinization, although it may only occur under relatively high concentrations and without trade‐offs in growth, development, or behavior.

## Graphical abstract

The proportion of wood frogs alive across nine populations when exposed to a lethal concentration of NaCl. Warmer colors indicate populations whose source pond had higher chloride concentrations and cooler colors indicate populations whose source pond had lower chloride concentrations. The asterisk indicates significantly higher survival.

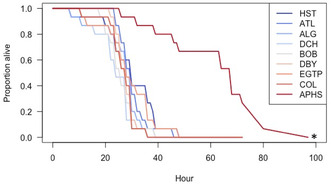

## 1 INTRODUCTION

The salinization of global freshwaters is a global issue with contributions from mining, agriculture, saltwater intrusion from sea level rise and storm surge events linked to climate change, and the application of de‐icing salts on roads in regions that experience snow and ice (Dugan et al., 2017; Hintz & Relyea, 2019). Indeed, more than 18 million metric tons of de‐icing salts are applied to roads in the United States each winter (Jackson & Jobbágy, 2005). Much of these de‐icing salts wash into adjacent freshwater habitats (Corsi et al., 2010; Dugan et al., 2017), which has myriad ecological consequences for aquatic species, communities, and ecosystems (Hintz & Relyea, 2019; Schuler et al., 2019). During the past decade, scientists have taken an “evolutionary toxicology” approach to better understand how freshwater organisms respond to salinization (Bickham, 2011; Brady et al., 2017). This is especially true as evolution via natural selection to anthropogenic changes on contemporary timescales in other conservation contexts has gained greater appreciation (Stockwell et al., 2003; Kinnison et al., 2007). Indeed, evolved tolerance to de‐icing salts has been demonstrated in various taxonomic groups including zooplankton, (Coldsnow & Relyea, 2018; Hintz et al., 2019) and aquatic macrophytes (Bora et al., 2020). However, it remains to be seen whether the ability to evolve tolerance to de‐icing salt pollution is common among freshwater organisms.

Given their narrow osmotic range (Boutilier et al., 1992), and sensitivity to chloride (Brady, 2012; Sanzo & Hecnar, 2006) amphibians have been of particular interest to scientists trying to understand the ability of organisms to adapt to the continuing stress of de‐icing salt pollution. Moreover, as amphibians are in decline globally (Stuart et al., 2004), understanding their ability to adapt to contaminants such as salts has important conservation implications (Blaustein & Bancroft, 2007). Some evidence suggests that it is possible for amphibians to evolve increased tolerance to high salt concentrations where they have likely lived in brackish habitats for centuries (Gomez‐Mestre & Tejedo, 2003; Hopkins & Brodie, 2015). However, freshwater salinization is a much more recent phenomenon, particularly in regions where de‐icing salts have been increasingly applied to roads since the 1940s. As a result, wetlands closer to roads can contain 20 times more salt than wetlands far from roads and these high salt concentrations can have substantial negative effects on the survival of amphibian embryos and larvae (Sanzo & Hecnar, 2006; Karraker et al., 2008; Brady, 2012; Petranka & Francis, 2013; Hill & Sadowski, 2016).

One amphibian species that has received growing research attention is the wood frog (*Rana sylvatica*), which has a geographic range that spans much of the eastern continental U.S. and Canada over to Alaska. Wood frogs are an excellent study species because they are explosive breeders, which results in populations throughout a region all breeding within the same week. This synchronized breeding behavior allows researchers to directly compare populations that are living in wetlands that vary widely in salinity. In past studies in Connecticut (USA), wood frog larvae that were collected as eggs from ponds with high salt concentrations exhibited lower survival (Brady, 2013, 2017), slower growth and development (Forgione & Brady, 2022), and reduced larval activity (Brady et al., 2022; Hall et al., 2017) compared to conspecific populations collected as eggs in wetlands far from roads. These studies have suggested that wood frogs are maladapted to salinization. However, other studies of wood frogs in Vermont (USA) have found that populations coming from high‐salt wetlands do not differ in survival, but have superior locomotor performance, adult mass, and fecundity, suggesting adaptation rather than maladaptation (Brady et al., 2019). Population‐level adaption to high‐salt concentrations has also been observed in a species of salamander in roadside ponds (*Ambystoma maculatum*; Brady, 2012), but contradictory results were found in short‐term lab studies (Brady et al., 2017).

It currently remains unclear what conditions seem to favor the evolution of adaptation versus maladaptation to salinization. Most studies showing maladaptation to salt have compared wood frog populations living in wetlands close to roads to wood frog populations living in wetlands far from roads (Brady, 2013, 2017). However, close proximity to roads (e.g., within 50 m, where salt concentrations are elevated; Karraker et al., 2008) may present additional selective forces (e.g., other pollutants) that may affect how populations are able to evolve adaptations or maladaptations to salt. An alternative approach would be to compare populations collected from wetlands that vary widely in salt concentration but are all in close proximity to roads.

In addition to examining adaptations and maladaptations to salinization by examining survival when exposed to lethal concentrations, it is also important to examine life history traits (e.g., growth, development) and behavior when animals are exposed to sublethal salt concentrations (Brady et al., 2022). This is particularly interesting when considering potential trade‐offs of evolved tolerance, where we might hypothesize that increased tolerance comes at the cost of slower growth, slower development, or impaired behavior. For example, wood frog populations can evolve tolerance to pesticides at the expense of parasite resistance (Billet et al., 2021; Hua et al., 2017).

To understand how amphibians may be evolving to salinization, we conducted two experiments using nine wood frog populations from wetlands that were all in close proximity to roads but differed widely in their salt concentrations. The first experiment tested the hypothesis that populations living in wetlands with high salt concentrations have evolved higher salt tolerance. The second experiment tested the hypothesis that populations that have evolved the highest salt tolerance will experience trade‐offs of slower growth, slower development, or impaired behavior.

## 2 METHODS

### 2.1 Animal collection

**FIGURE 1:**
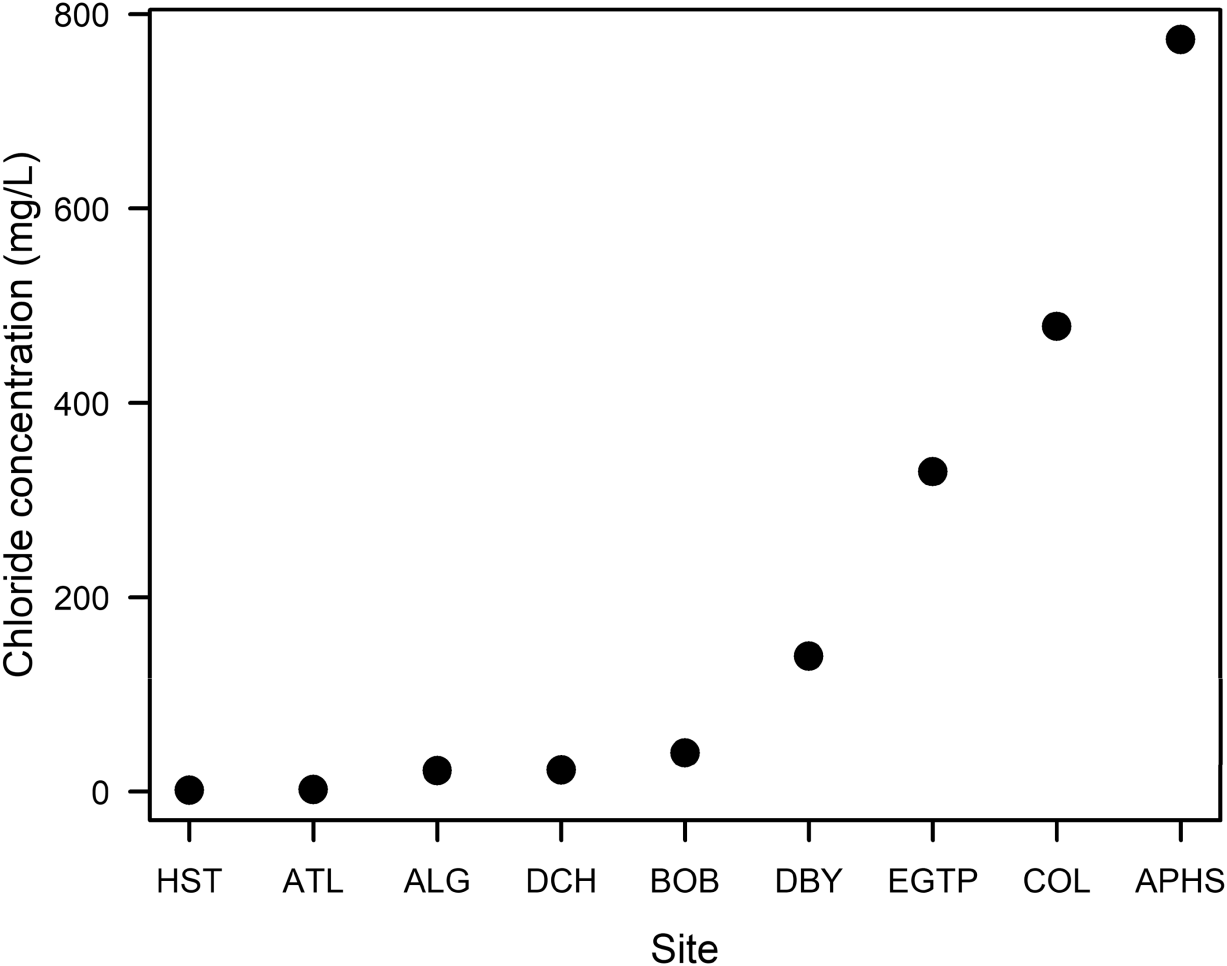
Chloride concentrations at each of the nine sites at the time of wood frog egg collection. Sites are represented by unique three‐ or four‐letter codes.

We collected newly oviposited wood frog egg masses from nine roadside ponds in upstate New York, USA that varied in their chloride concentrations at the time of egg collection, ranging from 1 to 744 mg Cl^−^/L (Figure 1). Given that salinity effects tend to diminish beyond 50 m (Karraker et al., 2008), all ponds were less than 40 m from the nearest road. During April 6–12, 2022, we gathered ten partial egg masses (i.e., approximately one‐fourth of a mass) from eight sites and seven partial masses from one site where only seven masses were laid. We placed the partial egg masses in 350‐L outdoor wading pools (one wading pool for each population) containing aged well water at the Rensselaer Aquatic Laboratory in Troy, NY. All populations began hatching between April 14 and 15, 2022. Once they hatched, we fed the tadpoles rabbit chow ad libitum (Blue Seal Fresh Show Hutch Deluxe). We used hatchling tadpoles from these egg masses in a time‐to‐death experiment followed by a growth and development experiment. At least 1 week prior to each experiment, we brought tadpoles from each population into the lab to acclimate. In the lab, the animal‐rearing room was maintained at 21°C with a light: dark cycle of 15:9 h.

### 2.2 Time‐to‐death experiment

To assess the relative levels of tolerance of each population to road salt contamination, we conducted a time‐to‐death (TTD) experiment where we exposed tadpoles to control water (19 mg Cl^−^/L) and a lethal concentration of 8 g/L of NaCl. Time‐to‐death experiments intentionally use high concentrations of a pollutant (often higher than experienced in nature) to determine whether different groups in individuals (e.g., populations) differ in their relative tolerance over a short time period (1–4 days), during which the animals are not fed (Brady, 2013; Bridges & Semlitsch, 2000; Semlitsch et al., 2000). Once TTD experiments find differences in tolerance based on short‐term times to death, researchers can examine environmentally relevant concentrations over longer periods of time to determine if differences in tolerance affect performance.

For this experiment, we used a fully randomized design that involved the nine populations of wood frogs and the two salt treatments. We placed one tadpole into each of the experimental units, which were 59 mL clear plastic cups filled with approximately 45 mL of water. The lethal salt dose was created by adding lab‐grade NaCl to aged tap water to reach a concentration of 8 g/L NaCl, based on the concentration used by Buss et al. (2021). From each population, we placed 15 individuals into cups containing water with a lethal salt concentration and five individuals into cups containing control water. Thus, there was a total of 180 cups in a completely randomized design. The control water was tap water that we aged at least 24 h to allow any chlorine to off‐gas. We also set aside ten individuals from each population to estimate the initial average mass and developmental stage (Gosner, 1960) of each population. The initial individual masses of the populations ranged from 33 to 64 mg; developmental stages ranged from approximately 26 to 27 (Table 1).

**TABLE 1:** The mean (±SE) initial mass and median Gosner developmental stage of wood frog tadpoles from each population were used in the time‐to‐death (TTD) and growth‐rate experiments.

Population	TTD experiment		Growth experiment	
	Mass (mg)	Gosner stage	Mass (mg)	Gosner stage
HST	46 ± 3	26	143 ± 8	28
ATL	52 ± 4	27	116 ± 8	27
ALG	60 ± 3	26	101 ± 9	27
DCH	56 ± 2	26	128 ± 9	28
BOB	33 ± 2	26	124 ± 7	27
DBY	45 ± 4	26	112 ± 5	27
EGTP	64 ± 2	27	125 ± 7	27
COL	48 ± 2	26	140 ± 6	28
APHS	43 ± 3	26	120 ± 4	27

*Note*: Site abbreviations are the same as in Figure 1.

We started the experiment on 26 April and team members took turns initially checking the tadpoles every 4 h for mortality. Once mortality began, we checked the experiment every 2 h and recorded the number of dead tadpoles from each population. We checked for deaths by gently blowing water at the tadpoles using a pipette. Nonresponsive tadpoles were considered dead. If it was unclear whether a tadpole was still alive, we looked for a heartbeat under a dissecting microscope. We set aside individuals who were declared dead and double checked them at the subsequent check time. If we determined that the tadpoles were still alive, we returned them to the experimental array. Checks continued for a total of 96 h (30 April). There was no mortality in the salt‐free controls. Upon completion of the experiment, we euthanized all individuals using 2% MS‐222.

We used pairwise log‐rank tests with a Bonferroni correction to look for differences in the Kaplan–Meier survival curves for each population. While this analysis tests for differences among the nine populations, it does not show what may be driving differences in survival. Thus, our analysis sought to determine the direct effect of the chloride concentration of the source pond and the death rates of each individual. To do so, we conducted a Cox mixed effects model. Our response variable in this model was the probability of tadpole survival, regardless of the pond from which they came. Our fixed effects were the source‐pond chloride concentrations, the mean individual mass of tadpoles in each population, and their interaction. We included population as a random effect in the model to control for other differences among populations, aside from chloride concentration, that we did not measure. We calculated hazard ratios for the fixed effects using the model parameter estimates. To do this, we raised the natural root to the power of the parameter values. Subtracting the hazard ratio from one gives the percent increase in survival that a parameter contributes to the overall survival of individuals in the study. Parameter significance was determined using Wald Chi‐square analyses. All analyses were conducted in R version 4.1.0 using the *survival* (Therneau, 2021), *Survminer* (Kassambara, 2021), *coxme* (Therneau, 2020), and *car* packages (Fox & Weisberg, 2019).

### 2.3 Growth and development experiment

To determine whether evolved salt tolerance came with any growth or developmental tradeoffs, we conducted a follow‐up experiment on additional wood frog tadpoles from the same nine populations. We used a randomized block design for this experiment with three spatial blocks, where each block was a different shelf height in the lab. In each block, experimental units were assigned one of the nine populations and one of two salt treatments (control water with no salt added vs. a higher sublethal salt concentration). We replicated the 18 treatment combinations twice within each block, which provided six replicates across the entire experiment. Thus, there were 36 experimental units per block and a total of 108 experimental units.

The experimental units were 5‐L white plastic tubs containing 4 L of aged tap water. On 9 May, we placed six tadpoles into each tub. As with the TTD experiment, we set aside ten individuals from each population at the outset of the experiment to quantify the average developmental stage (Gosner, 1960) and mass of the individuals used in the experiment from each population. These initial masses ranged from 62 to 179 mg with development stages ranging from approximately 25 to 28 (Gosner, 1960; Table 1).

We aged all tap water for at least 24 h to allow chlorine to off‐gas. For the sublethal salt treatment, we added lab‐grade NaCl in aged tap water to reach a chloride concentration of 165.5 ± 0.3 mg Cl^−^/L (mean ± SE). This value represents the mid‐range of chloride values in the ponds from which we collected the wood frogs (Figure 1). The tap water in the no‐salt treatment had a background chloride concentration of 25.9 ± 0.3 mg Cl^−^/L (mean ± SE).

We fed the tadpoles a ration of Tetra goldfish food (Spectrum Pet Brands LLC) mixed with water ad libitum (starting at approximately 16.5 mg per tadpole per day and increasing up to approximately 22.2 mg per tadpole per day as the tadpoles grew) over the course of the experiment. We conducted water changes approximately every 4 days over the course of the experiment whenever the water appeared murky.

Nine days into the experiment, we quantified tadpole activity using scan sampling. Two observers made 10 observations each of every tub over the course of 75 min, which produced 20 observations of each tub. The observers slowly walked by the tanks and peered in so as not to disturb the tadpoles. For each tub, we recorded the number of individuals moving at a given moment. We conducted the observations prior to feeding, given that satiated tadpoles substantially reduce their activity to low levels. We used the mean percentage of individuals moving in each tub as our response variable. For the two tubs that had a tadpole not survive, we adjusted the percent activity to reflect the reduced number of tadpoles in that tub. Survival across the entire experiment was 99.7%.

The experiment was terminated after 22 days (31 May), when the tadpoles approached metamorphosis (i.e., Gosner stage 39). We then determined the mass and Gosner stage of each tadpole and used the mean values of each experimental unit as our response variables.

We used a linear mixed effects model to look for differences in the relative growth rate of the tadpoles from the nine different populations exposed to two salt treatments. Relative growth rate was calculated as the difference in the natural log of final individual mass and initial population mean mass, divided by the duration of the experiment (i.e., 22 days). Our model used chloride concentrations of the source ponds, salt treatment (salt or no salt), and the interaction between these variables as the fixed effects. We had a series of nested random effects in this model, including block, population, and tub, nested in that order. These random effects were in place to account for additional variation introduced by the blocks, the individual tubs, and other aspects of the natal ponds that we did not measure directly.

We looked at the changes in tadpole development using a mixed effects model almost identical to the one we used for relative growth rate. In this instance, the response variable was the change in the Gosner stage for each individual tadpole. We calculated the change in the Gosner stage by subtracting the average initial Gosner stage of a given population from the final, measured Gosner stage of each individual in the experiment. Our fixed and random effects for this model were identical to the model examining relative growth rate.

Finally, we used a binomial mixed effects model to look for differences in activity among the nine populations in the two salt treatments. In this analysis, our response variable was whether an individual was moving or not during each of the twenty observation periods, hence the binomial model. The fixed and random effects variables in this model were the same as in the growth and development analyses. For each of these analyses (relative growth rate, development, activity), we used Wald II Chi square analyses to determine the significance of the fixed effects (chloride concentration, salt treatment, or their interaction). We conducted all our analyses in R version 4.1.0 (R Core Team 2022) using the *lme4* (Bates et al., 2015) and *car* (Fox & Weisberg, 2019) packages.

## 3 RESULTS

### 3.1 TTD experiment

**FIGURE 2:**
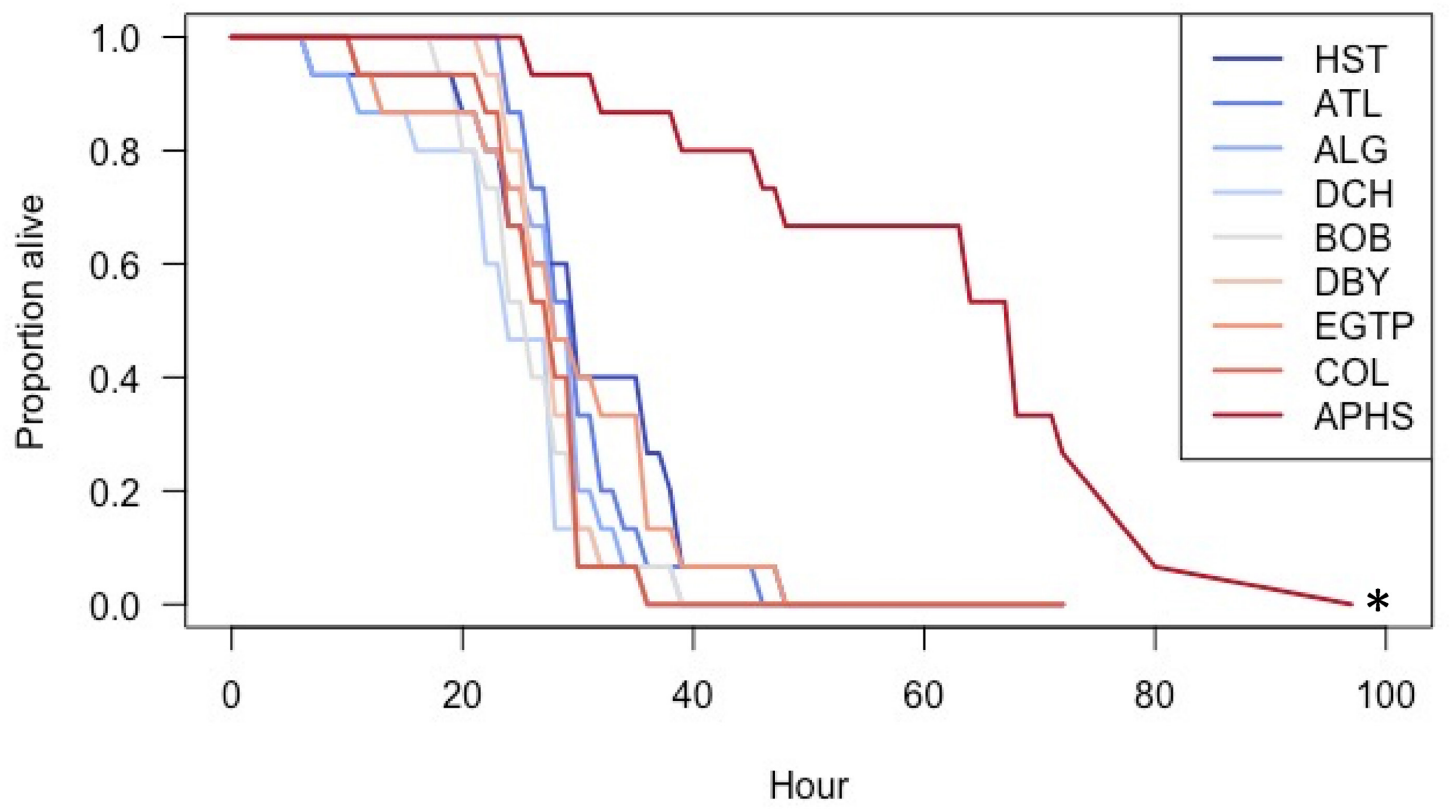
The proportion of wood frogs alive from each of the nine populations used in the time‐to‐death experiment when exposed to 8 g/L of NaCl. Warmer colors indicate populations whose source pond had higher chloride concentrations and cooler colors indicate populations whose source pond had lower chloride concentrations. The asterisk indicates significantly different Kaplan–Meyer survival as calculated using log‐rank tests (see text). There were no deaths in the no‐salt controls, so the survival curves for the nine controls are not shown.

The wood frogs from the APHS populations survived about twice as long in the lethal‐salt treatment compared to the other eight populations (Figure 2). Indeed, our log‐rank test showed that this group was statistically different from all the others (all pairwise *p* values for APHS < 0.01). The APHS population also had the highest concentration of chloride at the time of egg collection (Figure 1). The survival curves for the other eight the populations did not differ from each other (all pairwise *p* values > .2). Our Cox mixed effects model, designed to determine the explicit link between chloride concentration and survival, showed that chloride concentration had a significant, negative effect on the probability of dying in the TTD experiment (Table 2). In other words, individuals collected from ponds with higher chloride concentrations were more likely to survive than individuals collected from ponds with lower concentrations. However, this effect was quite weak, with a Hazard Ratio of more than 0.99 meaning that for wood frogs in our study, a single unit increase in chloride concentration confers less than a 1% increase in likelihood of survival Indeed, it is likely that this weak but statistically significant relationship is driven entirely by the APHS population.

**TABLE 2:** The results of a Cox mixed‐effects model examining chloride concentration of the source ponds, tadpole mass, and their interaction on tadpole survival in the time‐to‐death experiment.

Parameter	Estimate	Variation	HR	d.f.	${Χ}^{2}$	*p*
Pond chloride	−9.93 × 10^−3^	5.97 × 10^−3^	0.990	1	8.41	.004
Mass	−1.94 × 10^−2^	2.84 × 10^−2^	0.028	1	0.04	.834
Chloride × Mass	1.62 × 10^−4^	1.27 × 10^−4^	0.0001	1	1.65	.199
Population (random effect)	0.259	0.51	–	–	–	–

*Note*: The population was included in the model as a random effect. The estimate column shows the calculated parameter values for the fixed effects and the amount of variation explained by the random effect. The variation column shows the standard errors for the fixed effects and the standard deviation for the random effect of the population. HR is the hazard ratio, calculated by taking the exponent of the coefficient. Degrees of freedom, ${Χ}^{2}$ values, and *p* values were calculated using a Type II Wald ${Χ}^{2}$ analysis.

### 3.2 Growth and development experiment

**FIGURE 3:**
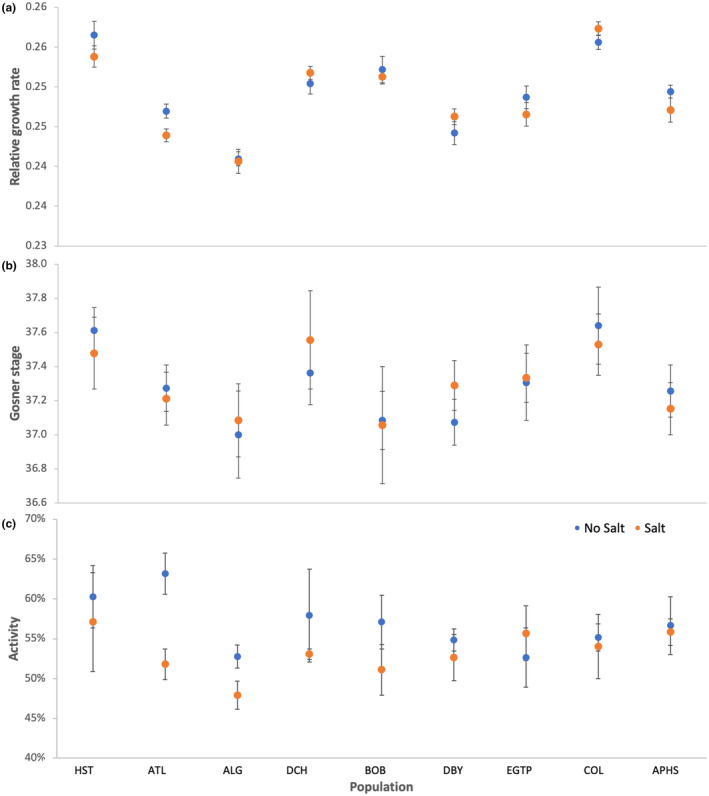
The (a) relative growth rate, (b) change in Gosner stage, and (c) activity of tadpoles for the nine wood frog populations in the presence and absence of a sublethal salt concentration. The populations are plotted in reference to the chloride concentration of their source ponds. Data points represent means ±1 SE.

While there were differences in the relative growth rates of the tadpoles (Table 3, Figure 3a), these differences were not explained by the chloride concentration of their natal pond, the salt treatments, or their interaction. Indeed, none of our model terms were significant and all effect sizes were small.

**TABLE 3:** ANOVA results of wood frog population and salt treatment on the relative growth rate, Gosner developmental stage, and activity of tadpoles.

Response variable	Explanatory variable	d.f.	*F*	*p*
Relative growth rate	Population (P)	8	33.65	<.001
	Salt (S)	1	1.49	.225
	P × S	8	1.33	.238
Gosner developmental stage	Population (P)	8	1.95	.62
	Salt (S)	1	0.01	.919
	P × S	8	0.214	.988
Activity	Population (P)	8	1.06	.40
	Salt (S)	1	5.00	.03
	P × S	8	0.74	.66

Similarly, while there were some differences in development among the nine populations (Table 3, Figure 3b), there were no significant effects on development for any of the variables we included in our models.

Tadpole activity was not affected by population, but was affected by the salt treatment (Table 3, Figure 3c). Across all populations, the moderate salt concentration made tadpoles less active. There was no interaction between population and salt treatment.

## 4 DISCUSSION

The tolerance, growth, development, and behavior of the wood frog tadpoles in our experiments were largely consistent between each of the nine populations with one notable exception. The population from the pond with the highest chloride concentration (APHS) showed a much higher tolerance to de‐icing salts than the other eight, as demonstrated by its higher survival in the lethal salt dose experiment. This finding supports our hypothesis that wood frogs from wetlands with higher salt concentrations can evolve higher salt tolerance.

Prior to this study, there was mixed evidence about the evolutionary responses of wood frogs to de‐icing salts. Our findings agree with Brady et al. (2019), who found that wood frog populations from Vermont exhibited superior locomotor performance and fitness‐related traits (e.g., body mass, fecundity) if they came from roadside ponds compared to populations from ponds far from roads. That study found no differences in survival between roadside versus woodland populations. However, it contrasted with studies of wood frog populations from Connecticut that have shown that de‐icing salts can lead to maladaptation (Brady, 2013, 2017). When studies have investigated the ability for wood frogs to adapt to roadside habitats with de‐icing salt pollution, they have compared roadside populations to those far from roads that receive no de‐icing salt pollution. Other work has shown that proximity to roads better explains egg density than salinity (Karraker et al., 2008) suggesting that for some maladapted traits, aspects of being near a road other than salinity may be important for determining the fitness of populations of wood frogs in these habitats. This could potentially explain why nearly all our populations showed identical responses in their growth and activity levels. That is, the evolved responses of wood frogs to de‐icing salts may also be affected by factors associated with roadside ponds.

One potential explanation for the pattern in our data is that the APHS population was only recently established and therefore had not evolved maladaptation the ways that the other eight populations had. In some populations of wood frogs, individuals from ponds far from roads have higher survival in high salt environments than those from roadside ponds (Brady, 2013, 2017; Brady et al., 2022; Forgione & Brady, 2022; Hall et al., 2017). However, it remains uncertain how many generations it takes for maladaptation to develop in wood frog populations living adjacent to roads. Rapid evolution, including adaptation and maladaptation, still requires multiple generations for trait change to occur (Hendry, 2017). However, this explanation would assume that maladaptation is the norm across all populations which isn't the case (Brady et al., 2019).

An alternative explanation is that the APHS population has evolved higher tolerance to de‐icing salts due to the higher concentration of salt in their source pond. Whether the increased tolerance we observed is the product of true evolution or phenotypic plasticity (e.g., Hua et al., 2015) is an open question. Regardless, the fact that we only saw increased tolerance in the population coming from the pond with the highest chloride concentration would suggest that there may be some threshold level of chloride pollution necessary for wood frogs to respond. Our highest salt concentration (774 mg Cl^−^/L) is similar to the highest concentrations observed in other studies, yet several of those studies did not detect increased tolerance in the highest salt populations (Brady & Goedert, 2017; Brady et al., 2017). However, as noted earlier, a subsequent study in Vermont did detect superior fitness‐related traits in roadside populations living with elevated salt concentrations (Brady et al., 2019).

Alternatively, the higher chloride tolerance of the APHS population could be the result of phenotypic plasticity. While all the tadpoles from each population in our experiments underwent the same exposure to road salts, plastic responses can still be present in the form of inherited environmental effects, such as maternal effects (Rossiter, 1996). As with frequency dependent selection, our experiment was not designed to test for mechanisms such as maternal effects. However, in the future, combining laboratory and field experiments would be helpful for exploring this possibility further.

Source pond chloride concentrations and our experimental salt treatments had no effect on the growth or development of the wood frog tadpoles. However, the experimental salt treatment did depress activity levels regardless of source pond chloride levels. The reduced activity levels in response to experimental salt treatments align with other studies that have found similar results in wood frogs (Hall et al., 2017) and other amphibians (Squires et al., 2010), although such affects can change over ontogeny (Kearney et al., 2014). This finding further reinforces the idea that current salt exposure may be more important for determining behavioral responses than the historic saltiness of the natal pond.

Interestingly, the higher tolerance to de‐icing salts in the APHS population did not lead to differences in growth rate, development, or activity. This is surprising, as there are good reasons to believe that these traits should be either higher or lower in the APHS population compared to the others. For example, it would make sense that having a higher tolerance to de‐icing salts would coincide with faster growth rates and higher activity levels, as shown in other studies (Hall et al., 2017). Alternatively, it would not have been surprising to see a tradeoff in growth and development rates with increased environmental stress (Berven et al., 1979). Indeed, tradeoffs associated with tolerance to pollutants have been recorded in wood frogs in other contexts (Hua et al., 2017). However, the growth and activity levels of the populations were not explained by the concentration of chloride in their natal ponds. It's possible that tradeoffs exist along other trait axes that we did not measure, such as parasite resistance, which has been shown to decrease with increasing chloride concentrations (Milotic et al., 2017). However, given the myriad of ways that maladaptation to de‐icing salts has been recorded in wood frogs, we must be careful to design studies that accurately assess whether trait changes are the result of tradeoffs or direct selection pressures (Schluter et al., 1991).

Unfortunately, we do not have historical records of the chloride concentrations in any of the ponds we used in our experiment, but it seems likely that similar concentrations have been occurring for decades. While the constituent sodium and chloride ions of de‐icing salts are typically conserved in waterbodies (Dugan et al., 2017; Godwin et al., 2003), they can fluctuate, especially since de‐icing salt application is not always consistent due to variation in weather patterns (Venäläinen, 2001). While we collected wood frogs from ponds that had a gradient in chloride concentrations at the time of collection, it is possible that these concentrations could experience springtime variation over multiple years.

That the population of wood frogs from the pond with the highest de‐icing salt pollution showed the highest tolerance was encouraging from a conservation perspective. However, it is important that we exercise caution when extrapolating short‐term, high‐concentration salt exposures to longer‐term, lower‐concentration experiments in nature, because sometimes one can observed contradictory outcomes, as observed in larval *Ambystoma* salamander populations (Brady, 2012; Brady et al., 2017). Moving forward, it will be necessary to begin focusing research efforts on populations of wood frogs from ponds with very high levels of chloride contamination (i.e., greater than 700 mg/L). Indeed, our most contaminated pond had a chloride level of 774 mg/L which is higher than many of the ponds used in previous studies (Brady, 2013, 2017). Focusing on highly impacted ponds will not only help determine whether our observation is part of a broader pattern, but also provide insight into the mechanisms that might drive higher tolerance to chloride among wood frog populations.

### 4.1 Conclusions

The results of this study demonstrate that populations of amphibians, and perhaps many other taxa, have the ability to evolve increase salt tolerance when exposed to high concentrations of salt in ponds and wetlands. Thus, we may need to focus our research efforts on populations of from ponds with very high levels of chloride contamination (i.e., greater than 700 mg/L). If such evolution of increased salt tolerance is common in aquatic taxa, it suggests that entire communities may have the ability to evolve higher tolerance, which would allow these species to persist while we work to reduce freshwater salinization, particularly through more targeted applications of road salts that can reduce salt use by as much as 50 percent (Hintz et al., 2022). Future studies should examine many other populations of amphibians and associated taxa and also examine whether the tolerance to salinization is constitutive or if it can be rapidly induced, as has been observed in many pesticides (e.g., Hua et al., 2015).

## AUTHOR CONTRIBUTIONS

**Rick Relyea:** Conceptualization (equal); data curation (equal); formal analysis (equal); funding acquisition (equal); investigation (equal); project administration (equal); supervision (equal); writing – original draft (equal); writing – review and editing (equal). **Brian Mattes:** Investigation (supporting). **Candace Schermerhorn:** Investigation (supporting). **Iasaac Shepard:** Conceptualization (equal); formal analysis (equal); investigation (lead); methodology (equal); supervision (equal); writing – original draft (equal).

## FUNDING INFORMATION

Major funding was provided by NSF grant DEB 16–55168 and Rensselaer Polytechnic Institute endowed chair funds.

## CONFLICT OF INTEREST STATEMENT

The authors declare no conflicts of interest.

## Supporting information


Data S1.

## Data Availability

All data will be publicly archived upon publication acceptance.
