# Supplementary material for: Freshwater salinization and the evolved tolerance of amphibians
Source: Ecol Evol. 2024 Mar 12;14(3):e11069. doi: 10.1002/ece3.11069 (PMC10933534; doi:10.1002/ece3.11069)
Supplement: Supplementary file 1 — Data S1. [file ECE3-14-e11069-s001.docx]

**Supplementary Material**

Table S1. The mean (± SE) initial mass and median Gosner developmental stage of wood frog tadpoles from each population used in the time-to-death (TTD) and growth-rate experiments. Site abbreviations are the same as in Fig. 1.

|  | TTD Experiment | | Growth Experiment | |
| --- | --- | --- | --- | --- |
| Population | Mass | Gosner stage | Mass | Gosner stage |
| HST | 46 ± 3 | 26 | 143 ± 8 | 28 |
| ATL | 52 ± 4 | 27 | 116 ± 8 | 27 |
| ALG | 60 ± 3 | 26 | 101 ± 9 | 27 |
| DCH | 56 ± 2 | 26 | 128 ± 9 | 28 |
| BOB | 33 ± 2 | 26 | 124 ± 7 | 27 |
| DBY | 45 ± 4 | 26 | 112 ± 5 | 27 |
| EGTP | 64 ± 2 | 27 | 125 ± 7 | 27 |
| COL | 48 ± 2 | 26 | 140 ± 6 | 28 |
| APHS | 43 ± 3 | 26 | 120 ± 4 | 27 |
